# YTHDF2-mediated m^6^A modification regulates mRNA stability of *Immediate early response gene 3* to modulate cell death in *Staphylococcus aureus-*induced bovine mastitis

**DOI:** 10.3389/fcimb.2025.1542647

**Published:** 2025-05-30

**Authors:** Yue Xing, Siyuan Mi, Siqian Chen, Xinyue Tao, Zihan Zhang, Yuanjun Shi, Xingping Wang, Ying Yu

**Affiliations:** ^1^ State Key Laboratory of Animal Biotech Breeding, National Engineering Laboratory for Animal Breeding, Breeding and Reproduction of Ministry of Agriculture and Rural Affairs, College of Animal Science and Technology, China Agricultural University, Beijing, China; ^2^ School of Life Sciences, Westlake University, Hangzhou, Zhejiang, China; ^3^ College of Animal Science and Technology, Ningxia University, Yinchuan, China

**Keywords:** bovine mastitis, *Staphylococcus aureus*, YTHDF2, m^6^A modification, apoptosis and necrosis

## Abstract

**Background:**

*Staphylococcus aureus* (*S. aureus*)-induced bovine mastitis is a major challenge for dairy production, causing significant economic losses. The regulatory mechanisms underlying host cell apoptosis and inflammation during *S. aureus* infection remain unclear. Therefore, this study investigates the role of N6-methyladenosine (m^6^A) modification and its reader protein YTHDF2 in regulating mRNA stability, apoptosis, and inflammation in bovine mammary epithelial cells (Mac-T cells) under *S. aureus* challenge.

**Methods:**

MeRIP-seq, RIP-seq, and RT-qPCR were used to analyze m^6^A-modified *IER3* mRNA and its interaction with YTHDF2. Apoptosis, necrosis, and mitochondrial function were assessed using YO-PRO-1/PI staining and JC-1 assays.

**Results:**

*S. aureus* infection significantly downregulated *YTHDF2* expression in Mac-T cells, leading to destabilization of m^6^A-modified *IER3* mRNA. This resulted in increased reactive oxygen species (ROS) levels, mitochondrial dysfunction, and cell apoptosis. Overexpression of *YTHDF2* restored mRNA stability, reduced apoptosis, and preserved mitochondrial function.

**Conclusion:**

YTHDF2 regulates m^6^A-modified mRNA stability to modulate apoptosis and inflammation during *S. aureus* infection. These findings provide new insights into understanding the molecular mechanisms of bovine mastitis and provide genetic markers for breeding mastitis-resistant dairy cows.

## 1 Introduction

Bovine mastitis, particularly caused by *Staphylococcus aureus* (*S. aureus*), represents a significant challenge to the dairy industry, resulting in reduced milk production, poor milk quality, substantial economic losses, and compromised animal welfare (Bannerman, 2009; Kang et al., 2016). One of the key pathological events in *S. aureus*-induced mastitis is the apoptosis of bovine mammary epithelial cells, which are the first line of defense against pathogens, triggering immune recognition and coordinating subsequent immune responses (Aitken et al., 2011). Understanding how *S. aureus* induces apoptosis in these cells, such as in Mac-T cells, is critical to developing genetic markers for breeding udder health in dairy cows (Wang et al., 2019; Lin et al., 2021; Ogunnaike et al., 2021).

N6-methyladenosine (m^6^A) is the most abundant RNA modification in eukaryotes and is regulated by methyltransferases, demethylases, and reader proteins (Jiang et al., 2021; Mao et al., 2023; Zhang et al., 2023a). It plays an essential role in regulating key biological processes, including cell differentiation, stress responses, and inflammation (Liu et al., 2020; Su et al., 2023; Zhu et al., 2023). While m^6^A’s involvement in bacterial infection-induced inflammation has been recognized, its specific mechanisms remain cell type- and pathogen-dependent (Zong et al., 2021; Prall et al., 2023; Liu et al., 2024). Recent studies have shown that m^6^A levels are significantly altered during *S. aureus* infection in mammary epithelial cells, suggesting that m^6^A may be critical in regulating the host cell immune response. However, its precise role in *S. aureus*-induced inflammation in bovine mammary epithelial cells remains poorly understood.

YTH N6-Methyladenosine RNA Binding Protein F2 (YTHDF2), an m^6^A reader, promotes mRNA degradation and is implicated in stress responses and inflammation (Guo et al., 2020; Zhang et al., 2022). However, its involvement in regulating immune responses during mastitis has not been fully explored. Given its role in mRNA stability and degradation, YTHDF2 may regulate immune responses in bovine mammary epithelial cells during *S. aureus* infection, and may play a critical role in modulating the inflammation and apoptosis observed in mastitis.

*Immediate early response gene 3* (*IER3*) (Yoon et al., 2009), also known as *IEX-1*, is an early response gene induced by various stress stimuli, including cytokines and DNA damage (Pawlikowska et al., 2010; Arlt and Schäfer, 2011). *IER3* is involved in apoptosis and cellular stress, interacting with key inflammatory signaling pathways such as NF-κB and PI3K/Akt (Arlt et al., 2003; Sebens Müerköster et al., 2008). Despite its relevance in various pathological conditions (Arlt et al., 2004; You et al., 2007), the precise role of *IER3* in *S. aureus*-induced apoptosis in mammary epithelial cells has not been fully elucidated.

This study investigates how YTHDF2-mediated m^6^A modification regulates *IER3* mRNA stability and its role in *S. aureus*-induced apoptosis in Mac-T cells. The findings of the current study will be beneficial for understanding the molecular mechanisms underlying mastitis and breeding disease resistance in dairy cows.

## 2 Materials and methods

### 2.1 Experimental design and samples information

**Figure 1:**
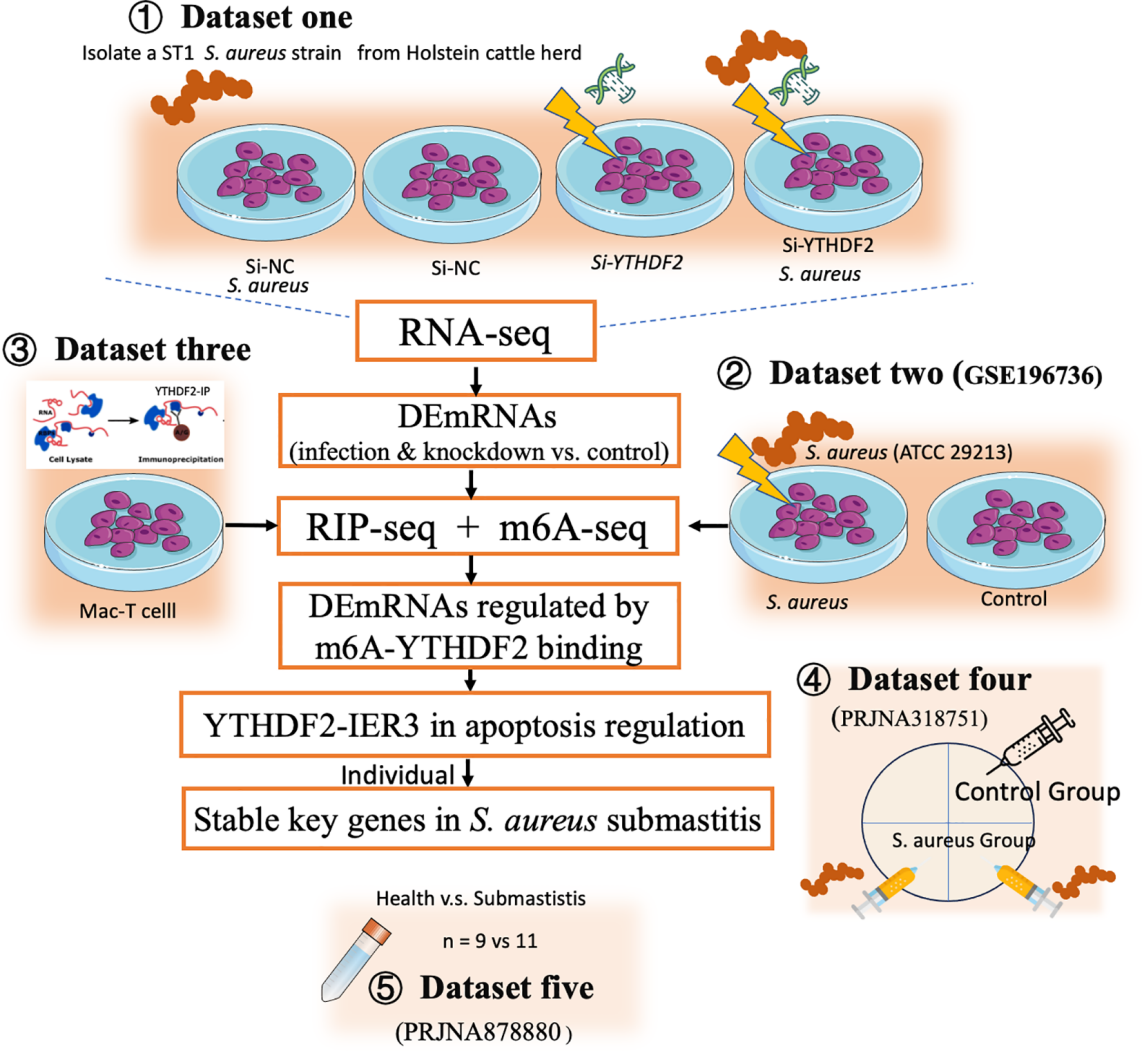
Technical routes of this study. This figure depicts the experimental design to investigate the role of YTHDF2-mediated m^6^A modification in regulating *IER3* mRNA stability during *S. aureus*-induced apoptosis. The study integrates both *in vitro* and *in vivo* approaches. *In vitro*, RNA-seq (Dataset one) and m^6^A-seq (Dataset two) were performed to assess gene expression and m^6^A modifications. RIP-seq (Dataset three) identified YTHDF2-bound mRNAs in Mac-T cells. Functional assays, including detection of apoptosis and oxidative stress, assessed the effects of YTHDF2 knockdown on *S. aureus* infection. *In vivo* validation was performed using publicly available RNA-seq datasets from bovine mammary gland tissue (Dataset four) and milk somatic cells from cows with subclinical mastitis (Dataset five).

This study aimed to investigate the role of YTHDF2-mediated m^6^A modification in regulating *IER3* mRNA stability during *S. aureus*-induced apoptosis, using both *in vitro* and *in vivo* strategies. The analyses combined data generated by the current study with publicly available datasets (**Figure 1**, **Table 1**).

**Table 1:** Overview of datasets used in this study.

Dataset ID	Dataset category	Sample type	Sample information	Condition
Dataset one	RNA-seq	The current study produced	Cell (Mac-T cell)	si-NC (*n* = 4) si-*YTHDF2* (*n* = 4) si-NC-*S. aureus* (*n* = 4) si-*YTHDF2*-*S. aureus* (*n* = 4)
Dataset two	m^6^A-seq	BioProject PRJNA675361 (GSE161050)	Cell (Mac-T cell)	Control-Input (*n* = 3) Control-m^6^A-IP (*n* = 3) *S. aureus-*Input (*n* = 3) *S. aureus*-m^6^A-Ip (*n* = 3)
Dataset three	RIP-seq	The current study produced	Cell (Mac-T cell)	Input (*n* = 3) YTHDF2-IP (*n* = 3)
Dataset four	RNA-seq	BioProject PRJNA318751	Individual (mammary gland tissue)	Control (*n* = 2) *S. aureus* (*n* = 2)
Dataset five	RNA-seq	BioProject PRJNA878880	Individual (milk somatic cell)	Healthy (*n* = 9) Submastistis (*n* = 11)

*In vitro*, Mac-T cells were divided into four experimental groups, including control (si-NC), *S. aureus* challenge (si-NC-*S. aureus*), YTHDF2 knockdown (si-YTHDF2), and YTHDF2 knockdown followed by *S. aureus* challenge (si-YTHDF2-*S. aureus*). RNA-seq was performed to assess gene expression differences between these groups (dataset one). To investigate the role of m^6^A modifications, m^6^A-seq (dataset two, publicly available) was used to compare *S. aureus*-infected cells with control cells, focusing on infection-specific m^6^A modifications. To identify YTHDF2-bound mRNAs, RIP-seq was performed in Mac-T cells (dataset three). This analysis specifically focused on characterizing the direct targets of YTHDF2 in uninfected cells, providing a baseline for understanding its regulatory role in mRNA stability. Functional assays, including YO-PRO-1/PI staining, ROS detection and JC-1 assay, were used to assess apoptosis, oxidative stress, and mitochondrial membrane potential in YTHDF2 knockdown cells during *S. aureus* infection.

*In vivo* validation of key gene expression was performed using RNA-seq datasets from bovine mammary gland tissues (dataset four, publicly available) and milk somatic cells from cows with subclinical mastitis (dataset five, publicly available). Regardless of their source, all RNA-seq datasets were processed uniformly to ensure comparability across experiments.

### 2.2 Cell culture and treatments

#### 2.2.1 Cell culture

Mac-T cells (bovine mammary epithelial cell line) were cultured in Dulbecco’s Modified Eagle’s Medium (DMEM) supplemented with GlutaMAX, 10% fetal bovine serum (FBS), and 100 U/mL penicillin-streptomycin (Thermo Fisher Scientific, USA). Cells were maintained at 37°C in a humidified incubator with 5% CO_2_ and passaged when they reached 80–90% confluence. Initially, cells were seeded in 25-cm² flasks (Corning), and upon reaching logarithmic growth, were transferred to 6-well plates (1 × 10^6^ cells/well), 12-well plates (1 × 10^5^ cells/well), 24-well plates (5 × 10^4^ cells/well), or 96-well plates (5 × 10³ cells/well) for further treatment.

#### 2.2.2 YTHDF2 small interfering RNA transfection

Mac-T cells seeded in 6-well plates at approximately 80% confluence were transfected with 100 pmol of YTHDF2-specific siRNA (Jintuosi, China) or negative control siRNA (si-NC) using 5 μL Lipofectamine 2000 (Thermo Fisher Scientific) in DMEM for 6 hours. Following transfection, the medium was replaced with fresh complete medium, and cells were cultured for an additional 40 hours before further treatment.

#### 2.2.3 Lentivirus packaging and generation of stable cell lines

Lentiviral particles containing YTHDF2 or control vector pNull (Jintuosi, China) were packaged in HEK293T cells by transfecting 3 μg of YTHDF2 plasmid, 3 μg of pMD2.G, and 6 μg of psPAX2 (Lipofectamine 2000, Invitrogen) in Opti-MEM I medium (Gibco). The viral supernatants were collected at 48 and 72 hours post transfection, filtered, and concentrated using Lenti-X Concentrator (Takara Bio).

Mac-T cells were seeded in 6-well plates, and after 24 hours, were infected with lentivirus (25 μL/mL) in the presence of 10 ng/mL polybrene (Sigma-Aldrich). After 48 hours, stable cells were selected using 4 μg/mL puromycin (InvivoGen) for 2 weeks and then maintained in 1 μg/mL puromycin to generate YTHDF2-overexpressing (oe-YTHDF2) cells.

### 2.3 Cow module sample preparation and treatment

Dataset four was derived from a previously published experiment conducted by our research group, involving two early lactating Holstein cows (Fang et al., 2016). Briefly, mastitis was induced by intramammary infusion of *S. aureus* suspensions (1 × 10^6^ CFU/mL) into the rear quarters immediately after morning milking, while the front quarters served as controls and received 10 mL sterile saline. Rectal temperature and somatic cell count (SCC, an indicator of leukocyte infiltration and immune response in milk) were measured at baseline (0 hours) and at 6, 12, 18, and 24 hours post infection (**Supplementary Table 1**). Inflammation onset was defined as SCC > 200,000 cells/mL and rectal temperature above 39.5°C. At 24 hours post infection, mammary gland tissues (udder biopsies) were collected from infected and control quarters, and RNA was extracted for subsequent RNA-seq analysis.

Dataset five consists of publicly available RNA-seq data from milk somatic cells of cows with subclinical mastitis and healthy control cows (Wang et al., 2024). Cows with subclinical mastitis were selected based on consistently high somatic cell counts (SCC > 350,000 cells/mL) over a period of three or more months, while healthy control cows had SCC < 100,000 cells/mL. The subclinical mastitis group included cows that tested positive for S. aureus in at least one quarter, while the healthy control group consisted of cows with no evidence of mastitis pathogens.

### 2.4 *S. aureus* culturing and challenge

The *S. aureus* strain used in this study is a sequence type 1 (ST1) isolate previously obtained and characterized by our laboratory from milk samples of cows with clinical mastitis (Wang et al., 2014b). Detailed information regarding its virulence factors is summarized in **Supplementary Tables 2**, **3** and has been described previously (Xing et al., 2025).

*S. aureus* was cultured overnight in tryptic soy broth (TSB) at 37°C with shaking at 200 rpm. Bacterial cultures were harvested by centrifugation at 5000 rpm for 5 minutes at room temperature, washed twice with sterile phosphate-buffered saline (PBS), and resuspended in PBS at a final concentration of 1 × 10^9^ CFU/mL.

For cell infection, Mac-T cells prepared were challenged with *S. aureus* at a multiplicity of infection (MOI) of 10:1, reaching a final bacterial concentration of 1 × 10^7^ CFU/mL per well. After incubation at 37°C and 5% CO_2_ for 6 hours, cells were gently washed with sterile PBS, harvested, and stored at -80°C for subsequent RNA extraction.

### 2.5 RNA extraction, library preparation, and RNA-seq analysis

#### 2.5.1 RNA extraction and library preparation

Total RNA was extracted from Mac-T cells using TRIzol reagent (Invitrogen). RNA purity and concentration were measured using a NanoDrop 2000 spectrophotometer (ThermoFisher Scientific, USA). RNA samples were stored at -80°C until use. For RNA-seq, 1 μg of total RNA was reverse-transcribed into cDNA using the PrimeScript RT reagent Kit (Takara, Kyoto, Japan). RNA-seq libraries were constructed and sequenced on the Illumina HiSeq2500 platform (Novogene Co., Ltd., Beijing, China), generating 150 bp paired-end reads.

#### 2.5.2 Data preprocessing and mapping

Publicly available RNA-seq datasets (datasets four and five) were downloaded from the SRA database using SRA-Toolkit v2.9.6. All datasets were processed uniformly. Raw read quality was assessed using FastQC v0.11.8, and reads containing adaptors, low quality bases, or undetermined bases were filtered using NGSQCToolkit v2.3.3. Clean reads were then mapped to the bovine reference genome (ARS-UCD1.2) using Hisat2 v2.1.0. SAM files were converted to BAM files with SAMtools v1.9, and read counts were quantified using FeatureCounts (subread v1.6.3).

#### 2.5.3 Normalization and differential expression analysis

Read counts obtained from FeatureCounts were normalized using DESeq2 v1.28.1 to generate normalized read counts across datasets. Differentially expressed genes (DEGs) were identified using DESeq2, with criteria of |log_2_FC| > 0.58 and p < 0.05. Comparisons included YTHDF2 knockdown vs. control (siYTHDF2 vs. siNc group) and *S. aureus* challenge vs. control (*S. aureus* vs. siNc group). Common DEGs between these comparisons were considered as key YTHDF2 target genes in bovine *S. aureus* mastitis.

### 2.6 N6-methyladenosine sequencing analysis

m^6^A sequencing data for Mac-T cells challenged with *S. aureus* and control cells were downloaded from the publicly available dataset GSE161050 (Li et al., 2021b). In study by Li et al. (2021b), Mac-T cells were co-cultured with heat-inactivated *S. aureus* (ATCC 29213) for 24 hours, after which RNA was extracted for m^6^A-seq analysis. Raw data were downloaded from the SRA database and converted to FASTQ format using SRA-Toolkit v2.9.6. 3. Sequence quality was assessed with FastQC v0.11.8, and reads containing adaptors, low quality bases, or undetermined bases were removed using NGSQCToolkit v2.3.3. Clean reads were then mapped to the bovine reference genome (ARS-UCD1.2) using Hisat2 v2.2.1, and SAM files were converted to BAM files using SAMtools v1.9. m^6^A peaks were called using the R package exomepeak2, with a q-value threshold of 0.05 to filter for significant peaks.

### 2.7 RNA-binding protein immunoprecipitation (RIP-seq) and analysis

RIP-seq was performed using the RNA Immunoprecipitation Kit (Bes5101, BersinBio, China) according to the manufacturer’s instructions. Briefly, Mac-T cells (1.0 × 10^7^, normal conditions) were lysed in RIP lysis buffer, with 15 μL reserved as input. The remaining 150 μL of lysate was incubated overnight at 4°C with anti-YTHDF2 antibody (Proteintech, Cat No. 24744-1-AP) or rabbit IgG-conjugated protein A/G magnetic beads in IP buffer with RNase inhibitors. Immunoprecipitated RNA was digested with proteinase K, purified using TRIzol reagent, and sequenced on the Illumina HiSeq 2500 platform (Novogene).

For data analysis, RIP-seq peaks were called using the same approach as for m^6^A-seq analysis. Peak calling was performed using the ChIPseeker package, and the chromosomal distribution of identified peaks was visualized. Motif analysis was then conducted on the common peaks shared between RIP-seq and m^6^A-seq using the HOMER software to identify potential RNA-binding motifs. These results were visualized using the Integrative Genomics Viewer (IGV, v2.8.0).

### 2.8 Quantitative real-time PCR and RIP-qPCR

Total RNA was reverse-transcribed into cDNA using the PrimeScript RT reagent kit (Takara, Japan). qRT-PCR was performed on the LightCycler 480 system with SYBR Green I Master Mix (Roche, Switzerland). GAPDH was used as a reference gene, and relative expression levels were calculated using the 2^–ΔΔCT^ method. RIP-qPCR specifically validated enrichment of *IER3* mRNA immunoprecipitated by YTHDF2 antibody under both normal and *S. aureus* challenged conditions, normalized against input RNA. Primer sequences are listed in **Supplementary Table 4**.

### 2.9 Western blot

Cell extracts were prepared using RIPA lysis buffer (Beyotime) with 1 mM PMSF and 1× protease inhibitor cocktail (Solarbio). Cells were lysed on ice for 30 minutes, then centrifuged at 12,000 rpm for 5 minutes at 4°C. Equal amounts of protein (20 µg) were loaded onto a 7.5% SDS-PAGE gel, separated, and transferred to PVDF membranes (Millipore, USA). Membranes were blocked with 5% milk in 1× TBST for 1 hour at room temperature, and then incubated with primary antibodies overnight at 4°C: YTHDF2 (1:3000, Proteintech, Cat No. 24744-1-AP) and GAPDH (1:3000, Abcam, ab8245). Membranes were washed three times with TBST (5 minutes each), followed by incubation with secondary antibodies for 1 hour at room temperature. Goat anti-rabbit secondary antibody (1:3000, Thermo Fisher Scientific, Cat No. 32460) was used for YTHDF2, and goat anti-mouse secondary antibody (1:3000, Thermo Fisher Scientific, Cat No. PA1-74421) was used for GAPDH. After three washes with TBST (5 minutes each), band signals were visualized using SuperKine UltraSensitive ECL substrate (Abbkine, BMU102).

### 2.10 Immunofluorescence

#### 2.10.1 Immunofluorescence staining

Mac-T cells were fixed with 4% paraformaldehyde for 15 minutes, permeabilized with 0.1% Triton X-100 for 10 minutes, and blocked with 5% normal goat serum (Vector) for 1 hour at room temperature. Cells were incubated with anti-YTHDF2 antibody (Proteintech, Cat No. 24744-1-AP) overnight at 4°C, followed by washing and incubation with a fluorescent secondary antibody (Thermo Fisher Scientific, Cat No. 32460) for 1 hour at room temperature. Nuclei were counterstained with DAPI (Sigma-Aldrich) for 5 minutes.

#### 2.10.2 Imaging and analysis

Images were captured using a ZEISS Axio Scope A1 microscope. Fluorescence intensity of YTHDF2 (488 nm) was compared with DAPI (405 nm), and relative expression levels were quantified using image analysis software.

### 2.11 Cell damage status analysis

Cell damage and viability were assessed after a 6-hour *S. aureus* challenge using assays for ROS detection, mitochondrial membrane potential (ΔΨm) analysis, and apoptosis (YO-PRO-1/PI staining).

#### 2.11.1 Intracellular reactive oxygen species detection

Intracellular ROS levels were assessed using DCFH-DA and DHE fluorescent dyes (Molecular Probes, Thermo Fisher Scientific) to specifically detect intracellular peroxides and superoxide anions, respectively. Briefly, cells were incubated with 10 μmol/L DCFH-DA for 20 minutes at 37°C, washed twice with PBS, and fluorescence intensity was quantified using a microplate reader at an excitation wavelength of 488 nm and an emission wavelength of 525 nm. ROS levels were expressed as relative fluorescence units (RFU), normalized to control groups.

#### 2.11.2 Mitochondrial membrane potential (ΔΨm) assessment

ΔΨm, an indicator of mitochondrial health and functionality, was assessed using JC-1 dye (Enzo Life Sciences), a fluorescent probe that shifts from red (aggregates, indicating high ΔΨm) to green fluorescence (monomers, indicating low ΔΨm) upon mitochondrial depolarization. After *S. aureus* challenge, cells were incubated with JC-1 staining solution (5 μg/mL) at 37°C for 20 minutes, washed with PBS, and observed under a fluorescence microscope. Fluorescence intensities were quantified, and mitochondrial depolarization was expressed as the ratio of green fluorescence intensity (monomers) to red fluorescence intensity (aggregates).

#### 2.11.3 Apoptotic cell staining

Apoptosis was assessed using YO-PRO-1/PI staining (Beyotime), a fluorescent method that distinguishes live, early apoptotic, and necrotic cells based on membrane integrity. YO-PRO-1 selectively enters early apoptotic cells and emits green fluorescence (excitation/emission: 491/509 nm), while propidium iodide (PI) penetrates necrotic cells and emits red fluorescence (excitation/emission: 535/617 nm). Cells were incubated with YO-PRO-1/PI staining solution for 20 minutes at 37°C, and fluorescence intensities were measured using confocal laser microscopy and a microplate reader.

### 2.12 mRNA stability assay

Cells were cultured to 50% confluence and treated with Actinomycin D (5 μg/mL). Samples were collected at 0, 3, and 6 hours after treatment. Total RNA was extracted and analyzed by RT-qPCR, with mRNA levels normalized to GAPDH. mRNA degradation rates were determined according to established protocols (Mukherjee et al., 2011; Wang et al., 2014a).

### 2.13 Function enrichment analysis

#### 2.13.1 Gene set enrichment analysis

GSEA v4.3.3 was used to identify significantly enriched pathways activated under *S. aureus* infection, YTHDF2 knockdown (siYTHDF2), and combined siYTHDF2-*S. aureus* treatment conditions. Genes were ranked by log_2_ fold-change from normalized expression data. Enrichment significance was assessed by 1,000 permutations, with enriched pathways defined by an FDR q-value < 0.05.

#### 2.13.2 Kyoto encyclopedia of genes and genomes pathway analysis

KEGG pathway analysis of DEGs was conducted using KOBAS 3.0 (http://bioinfo.org/kobas/). Pathways with a p < 0.05 were considered significantly enriched. Visualization of the top 20 pathways (p < 0.05) was performed using ImageGP and ggplot2.

#### 2.13.3 Quantitative trait loci enrichment analysis

QTL enrichment analysis, a genetic approach used to identify genomic regions associated with quantitative phenotypic traits, was conducted to identify cattle QTLs harboring DEGs and 44 YTHDF2-regulated genes involved in *S. aureus*-induced mastitis. Cattle QTL data were obtained from AnimalQTLdb (https://www.animalgenome.org/cgi-bin/QTLdb/index) (Hu et al., 2022). QTLs within 100 kb upstream or downstream of the target genes were screened.

#### 2.13.4 Transcriptome-wide association studies analysis

Trait associations were predicted using TWAS, an analytical method that integrates gene expression data with genome-wide association studies (GWAS) to identify genetic associations with specific phenotypic traits. The analysis used the cGTEx database (cgtex.roslin.ed.ac.uk) and integrated TWAS results with QTL data for the 44 candidate genes identified in this study.

#### 2.13.5 GWAS enrichment analysis

GWAS enrichment analysis used data from a University of Maryland and USDA collaborative project involving 27,143 dairy cattle (https://figshare.com/s/ea726fa95a5bac158ac1). The analysis focused on 44 complex traits related to production, reproduction, and health. SNPs within a 10 kb flanking region of each candidate gene were screened, and a hypergeometric test was used to assess the overrepresentation of significant SNPs. The Benjamini-Hochberg procedure adjusted for multiple testing, with an adjusted p < 0.05 considered statistically significant.

### 2.14 Statistical analysis

Statistical analyses were performed using R v4.0.5 and GraphPad Prism v9.0, and quantification of necrotic cell was performed using ImageJ v1.51. Student’s t-test was used to compare differences between *S. aureus* challenged and control groups. Data are presented as mean ± standard deviation (SD), with significance set at p < 0.05.

## 3 Results

### 3.1 *S. aureus*-induced inflammation in Mac-T cells increases apoptosis and necrosis while significantly reducing *YTHDF2* expression

**Figure 2:**
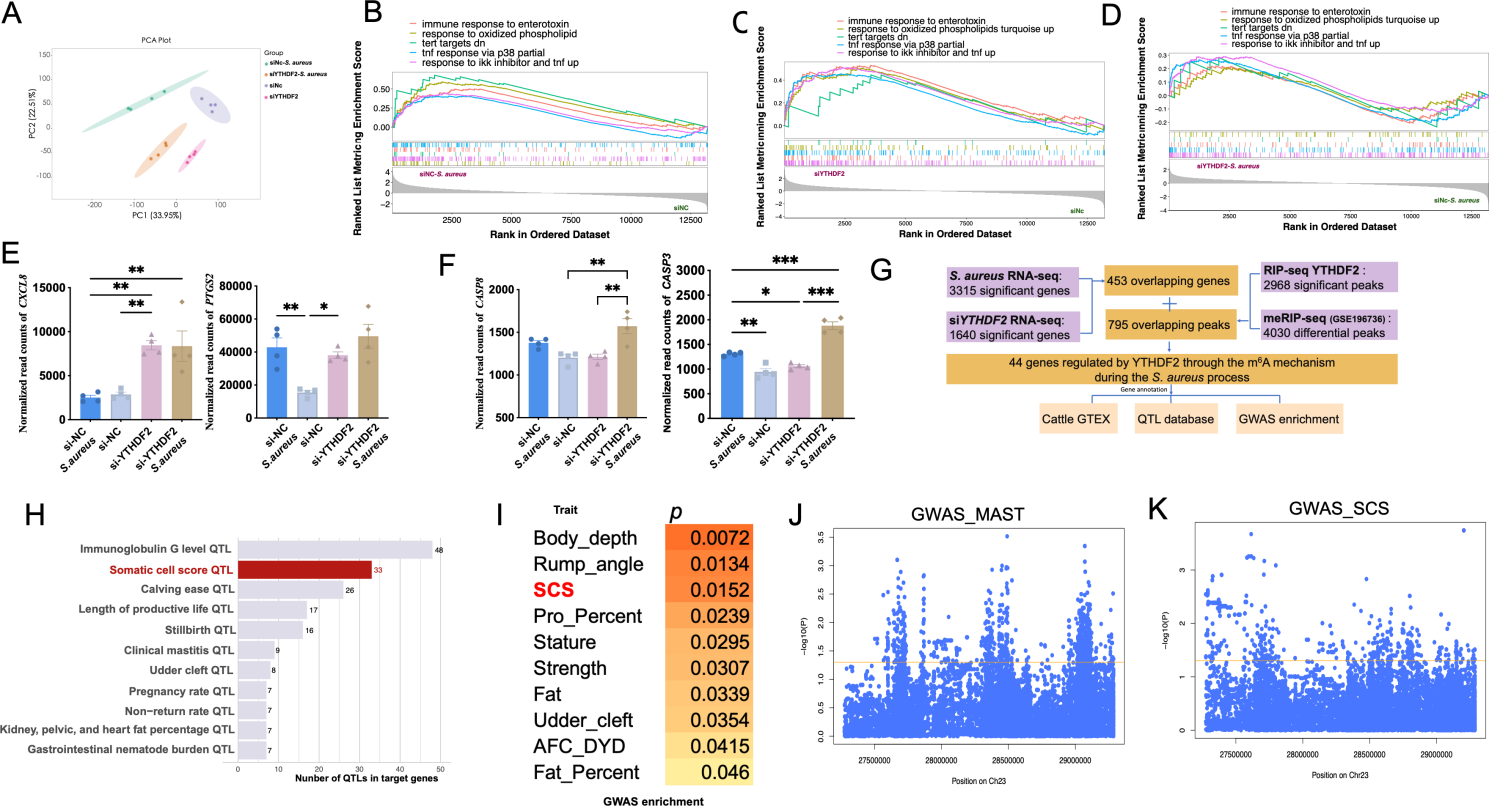
Functional impact of YTHDF2 binding on gene expression in Mac-T cells. **(A)** PCA of gene expression profiles in different experimental groups: si-NC, si-*YTHDF2*, si-NC + *S. aureus*, and si-*YTHDF2* + *S. aureus*. **(B)** GSEA comparing gene expression in the si-NC + *S. aureus* group to the si-NC group. **(C)** GSEA comparing gene expression in the si-YTHDF2 group to the si-NC group. **(D)** GSEA comparing gene expression in the si-YTHDF2 + *S. aureus* group to the si-NC + *S. aureus* group. **(E)** Expression levels of pro-inflammatory cytokines in different experimental groups, with statistical significance indicated: *p < 0.05, **p < 0.01, ***p < 0.001. **(F)** Expression levels of apoptosis marker genes in different experimental groups, with statistical significance indicated: *p < 0.05, **p < 0.01, ***p < 0.001. **(G)** Overlap between RIP-seq and MeRIP-seq data, showing genes regulated by YTHDF2 through m^6^A modification. **(H)** QTL analysis of the overlapping differentially expressed genes (DEGs), showing associations with health-related traits in cattle, including immunoglobulin G level, somatic cell score (SCS), and clinical mastitis. **(I)** GWAS enrichment analysis of the overlapping DEGs, highlighting associations with SCS in cattle. **(J, K)** Results of SNP analysis within ±1 Mb of IER3, showing associations with mastitis and SCS traits, with the yellow line representing the p = 0.05 threshold.

RNA-seq was performed on Mac-T cells from four experimental conditions, including siNC (negative control), siYTHDF2, siNC-*S. aureus*, and siYTHDF2-*S. aureus*. Principal component analysis (PCA) revealed a clear separation among these conditions, indicating distinct transcriptional profiles resulting from both *S. aureus* infection and YTHDF2 knockdown (**Figure 2A**).

Gene set enrichment analysis (GSEA) identified key pathways that were activated across the experimental conditions. In siNC-*S. aureus* cells, pathways associated with inflammation and apoptosis were significantly activated, including immune response to enterotoxin, response to IKK inhibitor and TNF signaling, response to oxidized phospholipids, TNF response via p38, and TERT targets down-regulated genes (**Figure 2B**). Notably, similar activation of these inflammatory and apoptotic pathways was observed in siYTHDF2 cells, even in the absence of *S. aureus* infection (**Figure 2C**), suggesting that YTHDF2 knockdown alone is sufficient to induce inflammation. When comparing siYTHDF2-*S. aureus* cells to siNC-*S. aureus* cells, these pathways remained significantly enriched, indicating that YTHDF2 loss exacerbates the inflammatory and apoptotic responses to *S. aureus* infection (**Figure 2D**).

Furthermore, pro-inflammatory factors, such as *CXCL8* and *PTGS2*, were significantly upregulated after YTHDF2 knockdown, with the highest expression levels observed in cells with both YTHDF2 knockdown and *S. aureus* infection (**Figure 2E**). Similarly, the apoptotic markers *CASP3* and *CASP8* were significantly upregulated in *S. aureus*-infected cells, with the highest expression levels detected in the *S. aureus*-infected YTHDF2 knockdown cells (**Figure 2F**).

### 3.2 *YTHDF2* regulates m6A-modified genes associated with bovine health traits during *S. aureus* infection

Differential expression analysis identified 3,315 differentially expressed genes (DEGs) (|log2FC| > 0.58, p < 0.05) between *S. aureus*-infected cells and control cells (**Figure 2F**). KEGG enrichment analysis of these DEGs revealed significant activation of apoptosis, TNF signaling, and inflammatory response pathways (**Supplementary Figure S1A**). A similar RNA-seq analysis comparing YTHDF2 knockdown cells and control cells also identified 3,315 DEGs (|log2FC| > 0.58, p < 0.05), enriched in pathways related to homologous recombination, apoptosis, and DNA repair (**Supplementary Figure S1B**). Of these, 453 overlapping genes were identified, enriched in pathways such as pyrimidine metabolism, PPAR signaling, and immune response regulation (**Supplementary Figure S1C**).

To investigate m^6^A modification during *S. aureus* infection, m^6^A-seq was performed using publicly available m^6^A-seq data (*S. aureus* infection vs. control). The analysis identified 4,030 distinct m^6^A methylation sites responsive to *S. aureus* challenge. To explore the potential regulatory role of YTHDF2 in m^6^A-modified genes, RIP-seq was performed using control cells. RIP-seq analysis revealed 10,284 peaks with a q-value < 0.05 across the genome and identified 2,968 significantly enriched transcripts (**Supplementary Figure S2A**).

Integrating the m^6^A-seq data with the YTHDF2 RNA-binding sites identified in this study, 795 genes with YTHDF2-dependent m^6^A modifications were obtained. Overlapping results confirmed the DRACH motif (D=A/G/U, R=A/G, H=U/A/C) as the canonical sequence for m^6^A modification (**Supplementary Figure S2B**). These findings emphasized YTHDF2’s role in recognizing and binding m^6^A-modified mRNAs, with significant implications for gene expression regulation in *S. aureus*-infected cells.

Cross-referencing the identified genes with the 453 overlapping DEGs, 44 candidate genes potentially regulated by YTHDF2-mediated m^6^A modification during inflammation were identified (**Figure 2G**, **Supplementary Figures S2, D**). Gene annotation based on the TWAS database highlighted significant associations between several of these candidate genes (such as *SPP1*, *FAM13A*, *NDRG1*, and *IER3*) and key bovine health traits, including milk protein percentage, body depth, and ketosis (a metabolic disease of dairy cow) susceptibility (**Table 2**). Further QTL analysis identified 288 health-related QTLs within ±100 kb of these candidate genes, particularly associated with immunoglobulin G levels, somatic cell score, and clinical mastitis (**Figure 2H**, **Supplementary Figure S2E**). GWAS enrichment analysis further confirmed significant enrichment of these candidate gene regions with genomic loci associated with somatic cell score, supporting the role of YTHDF2-mediated m^6^A modifications in regulating bovine health traits during *S. aureus* infection (**Figure 2I**).

**Table 2:** Trait prediction of candidate gene in TWAS data.

Gene	Tissue	Trait	*Z*score	*P*	pred_perf_*p*val
*SPP1*	Liver	Protein percentage	7.26	0.00	0.00
*FAM13A*	Blood	Protein percentage	-5.66	0.00	0.00
*CALHM2*	Adipose	Livabitity	2.93	0.00	0.00
*NDRG1*	Macrophage	Body depth	-2.87	0.00	0.03
*BTBD11*	Blood	Udder cleft	-2.21	0.03	0.00
*PROCA1*	Mammary	Teat length	3.52	0.00	0.00
*GRB7*	Blood	Protein	2.74	0.01	0.00
*IER3*	Adipose	Ketosis	2.09	0.04	0.00
*PPP1R15A*	Intramuscular fat	Protein	-2.01	0.04	0.00
*ARHGAP24*	Blood	Heifer conception rate	2.04	0.04	0.00
*TRMT5*	Lung	Sire conception rate	2.08	0.04	0.04
*KDM3A*	Macrophage	Fore udder attachment	2.36	0.02	0.05
*BTG1*	Blood	Milk	-2.84	0.00	0.00
*CSPG4*	Intramuscular fat	Sire still birth	-2.06	0.04	0.01
*NUMBL*	Blood	Ketosis	-2.21	0.03	0.00
*KLHL24*	Macrophage	Udder cleft	1.99	0.05	0.03
*ST8SIA5*	Muscle	Daughter pregnacy rate	-2.99	0.00	0.00
*FBXL3*	Milk cell	Sire calving ease	2.10	0.04	0.04

Zscore: S-PrediXcan’s association result for the gene, typically HUGO for a gene.

Pred_Perf_Pval: P-value of tissue model’s correlation to gene’s measured transcriptome (prediction performance).

Interestingly, *IER3* also showed an association with immunoglobulin G levels (**Supplementary Figure S2F**), suggesting a potential link between this gene and bovine health traits. Key SNPs associated with mastitis and somatic cell score (SCS, a standardized measure of SCC used to assess milk quality and udder health) traits in dairy cows are located within a 1 Mb region of the *IER3* gene, and *IER3* could be considered as a candidate gene for genetic selection of mastitis resistance (**Figures 2J, K**).

### 3.3 Knockdown and overexpression of *YTHDF2* reveals its role in *S. aureus*-induced apoptosis in Mac-T cells

**Figure 3:**
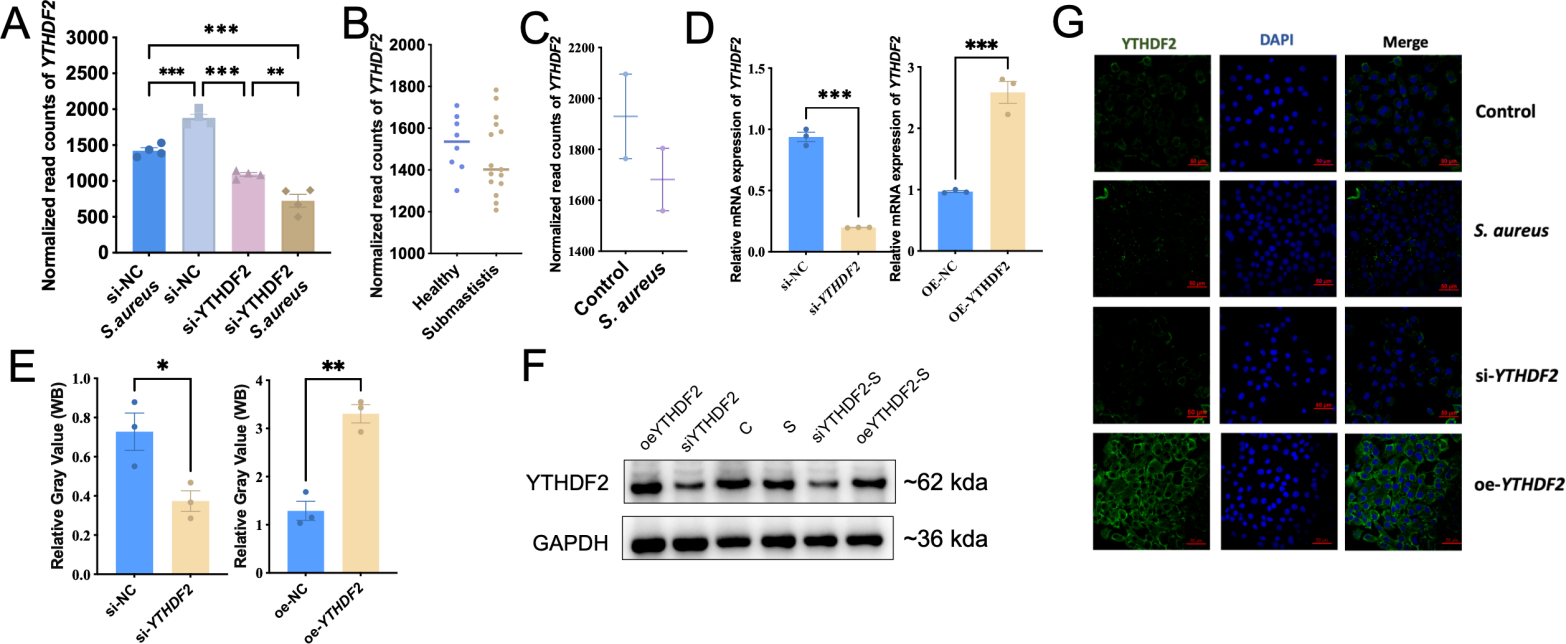
YTHDF2 expression in Mac-T cells under *S. aureus* challenge. **(A)** Normalized *YTHDF2* expression levels in Mac-T cells from different experimental groups: si-NC, si-*YTHDF2*, si-NC + *S. aureus*, and si-*YTHDF2* + *S. aureus*. **(B)** Normalized *YTHDF2* expression in milk samples from subclinical *S. aureus* mastitis cows and healthy cows. **(C)** Normalized *YTHDF2* expression in mammary gland tissue samples from the bovine model. **(D)** Relative mRNA expression of *YTHDF2* in Mac-T cells transfected with si-NC, si-*YTHDF2*, oe-NC, and oe-*YTHDF2*, as determined by RT-qPCR. **(E)** WB analysis showing YTHDF2 protein levels and relative gray values in Mac-T cells transfected with si-NC, si-YTHDF2, oe-NC and oe-YTHDF2. **(F)** WB showing YTHDF2 protein levels in different experimental groups. **(G)** Immunofluorescence images showing YTHDF2 expression (green), DAPI (blue), and merged images in control, *S. aureus*-challenged, si-*YTHDF2* and oe-*YTHDF2* Mac-T cells. Statistical significance is indicated as follows: *p < 0.05, **p < 0.01, ***p < 0.001.

Dataset one RNA-seq analysis showed a significant reduction in YTHDF2 expression in siNC-*S. aureus* cells compared to siNC controls, with a further reduction observed in siYTHDF2-*S. aureus* cells, indicating a synergistic effect of *S. aureus* infection and YTHDF2 knockdown (**Figure 3A**). This trend was further confirmed by transcriptomic data from milk samples of cows with subclinical mastitis and blood samples from cows infected with *S. aureus*, providing additional evidence for the downregulation of YTHDF2 during *S. aureus* infection (**Figures 3B, C**).

Given this observed reduction in YTHDF2 expression during *S. aureus*-induced mastitis, its potential regulatory role in apoptosis was investigated through both knockdown and overexpression experiments in Mac-T cells. Knockdown of YTHDF2 (si-YTHDF2) significantly reduced its expression at both mRNA and protein levels, as confirmed by RT-qPCR and Western blot analyses, whereas overexpression of YTHDF2 (oe-YTHDF2) resulted in a significant increase in both mRNA and protein levels (**Figures 3D, E**). Notably, *S. aureus* infection caused a further decrease in YTHDF2 expression, regardless of the experimental conditions, including both the si-YTHDF2 and oe-YTHDF2 groups (**Figure 3F**). Immunofluorescence assays confirmed the effective knockdown and overexpression of YTHDF2 protein in the respective experimental conditions (**Figure 3G**).

**Figure 4:**
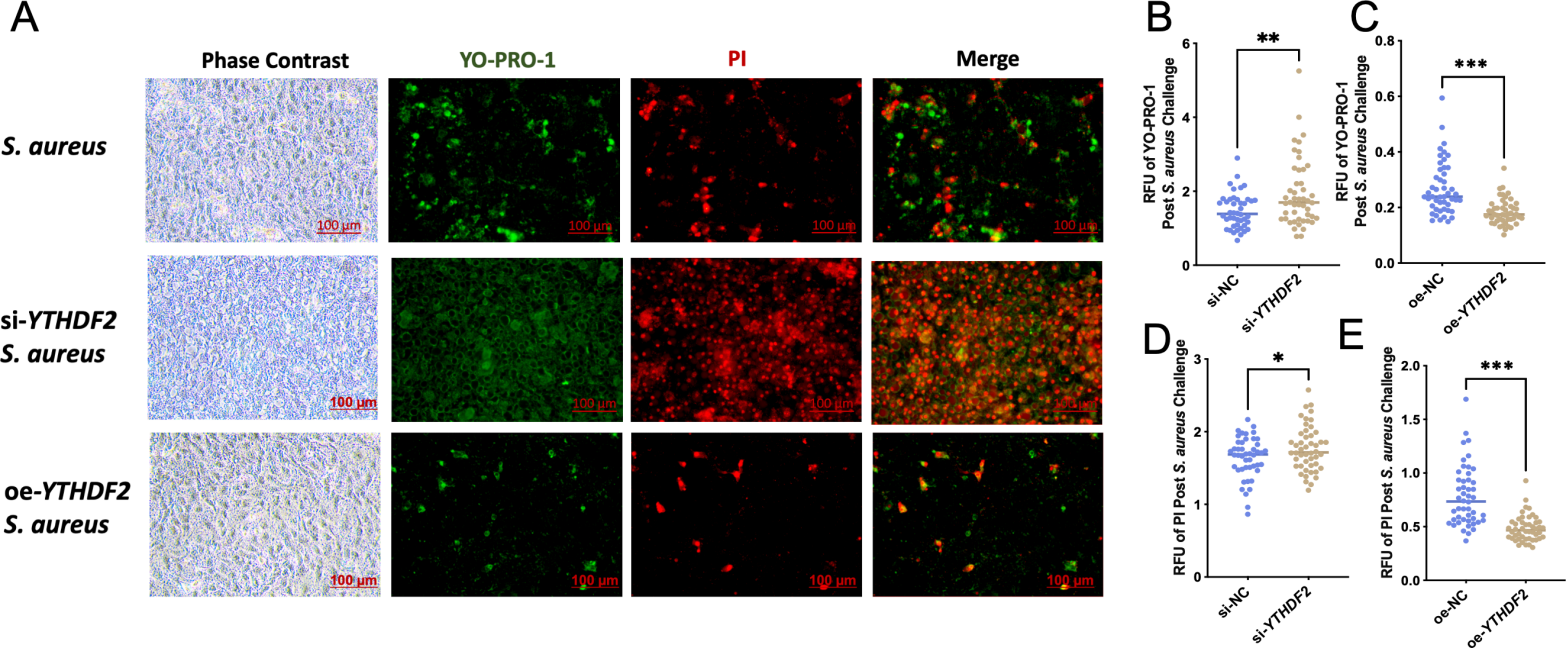
YTHDF2 modulates apoptosis in Mac-T Cells under *S. aureus* challenge. **(A)** Phase contrast and YO-PRO-1/PI staining images showing apoptosis and necrosis in *S. aureus*-challenged, si-*YTHDF2* and oe-*YTHDF2* Mac-T cells. Green fluorescence indicates apoptotic cells (YO-PRO-1) and red fluorescence indicates necrotic cells (PI). **(B-E)** Relative fluorescence units (RFU) of YO-PRO-1 and PI in si-NC, si-YTHDF2, oe-NC and oe-YTHDF2Mac-T cells post S. aureus challenge. Statistical significance is indicated as follows: *p < 0.05, **p < 0.01, ***p < 0.001.

Functional assays using YO-PRO-1/PI staining, which detects apoptosis and necrosis based on differential dye uptake, showed that YTHDF2 knockdown significantly enhanced *S. aureus*-induced apoptosis and necrosis compared to controls (**Figure 4A**). Conversely, cells overexpressing YTHDF2 showed significantly reduced apoptosis and necrosis following bacterial challenge (**Figure 4A**). These results were quantitatively validated by microplate reader measurements of YO-PRO-1 and PI fluorescence, where YTHDF2 knockdown resulted in significantly higher fluorescence intensity, whereas YTHDF2 overexpression resulted in lower fluorescence signals compared to controls (**Figures 4B–E**).

### 3.4 YTHDF2 modulates apoptosis through ROS-mediated mitochondrial dysfunction in Mac-T cells under *S. aureus* challenge

**Figure 5:**
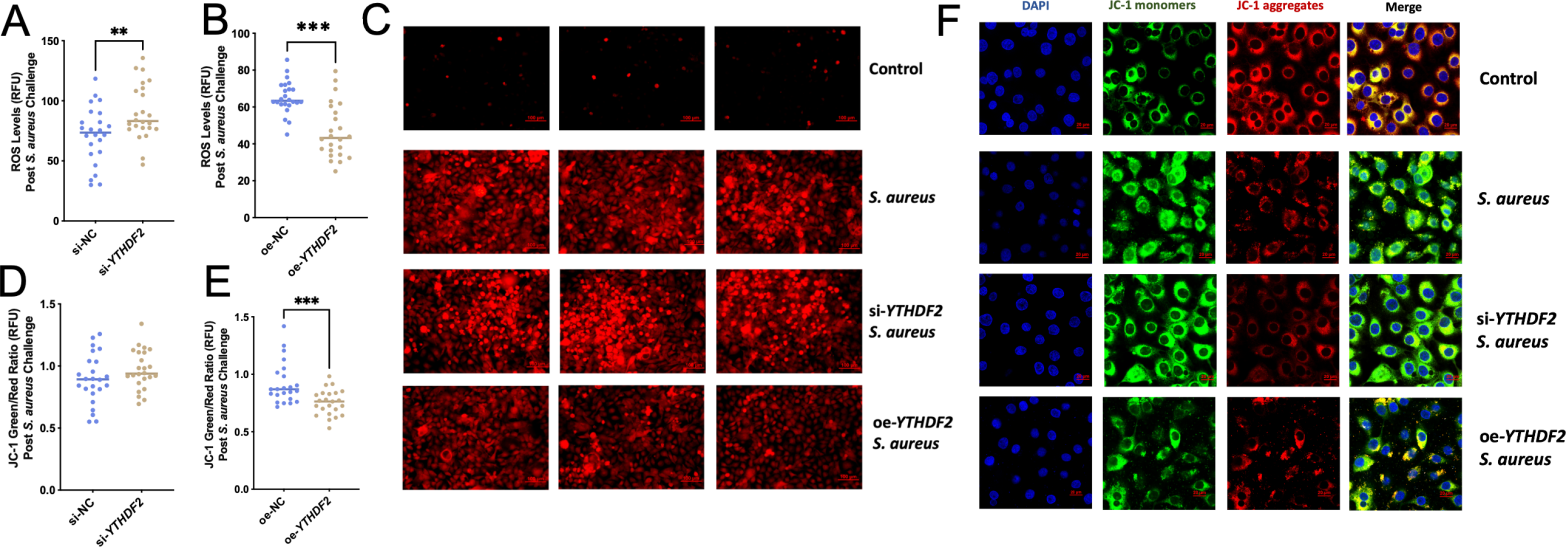
Effects of YTHDF2 on ROS levels and mitochondrial membrane potential in Mac-T Cells under *S. aureus* challenge. **(A, B)** RFU of ROS levels in si-NC,si-YTHDF2, oe-NC and oe-YTHDF2 Mac-T cells post S. aureus challenge. Statistical significance is indicated as follows: **p < 0.01, ***p < 0.001. **(C)** Fluorescent microscopy images showing ROS levels in control, *S. aureus*-challenged, si-*YTHDF2* and oe-*YTHDF2* Mac-T cells. **(D, E)** JC-1 green/red ratio indicating mitochondrial membrane potential (MMP) in si-NC, si-*YTHDF2*, oe-NC and oe-*YTHDF2* Mac-T cells post *S. aureus* challenge. **(F)** Confocal microscopy images showing JC-1 monomers (green), JC-1 aggregates (red), and merged images in control, *S. aureus*-challenged, si-*YTHDF2* and oe-*YTHDF2* Mac-T cells.

ROS are known to accumulate during oxidative stress and play a critical role in mediating apoptosis by disrupting mitochondrial function (Maassen et al., 2004; Rizwan et al., 2020). In this study, YTHDF2 knockdown (si-YTHDF2) significantly increased ROS levels in Mac-T cells following *S. aureus* infection compared to controls (**Figure 5A**), contributing to mitochondrial dysfunction. Conversely, overexpression of YTHDF2 (oe-YTHDF2) reduced ROS levels and preserved mitochondrial function in *S. aureus*-challenged cells (**Figure 5B**). The results of the zymography assay were further supported by fluorescence microscopy, which more clearly demonstrated changes in fluorescence intensity (**Figure 5C**).

Mitochondrial membrane potential (MMP) was analyzed using the JC-1 assay, as changes in MMP are a hallmark of apoptosis. In si-YTHDF2 cells, the green/red fluorescence ratio was significantly higher, indicating mitochondrial dysfunction (**Figure 5D**). In contrast, oe-YTHDF2 cells showed a lower green/red ratio, indicating preserved mitochondrial membrane potential (**Figure 5E**). These findings were further confirmed by confocal microscopy, which showed that si-YTHDF2 cells exhibited increased green fluorescence (MMP loss), whereas oe-YTHDF2 cells showed predominantly red fluorescence (intact MMP) (**Figure 5F**).

These results establish YTHDF2 as a key regulator of mitochondrial function in *S. aureus*-infected Mac-T cells. By modulating ROS levels and maintaining mitochondrial membrane potential, YTHDF2 protects cells from oxidative stress and apoptosis, highlighting its essential role in mitigating *S. aureus*-induced mitochondrial dysfunction and ensuring cellular homeostasis during mastitis.

### 3.5 *IER3* expression is significantly increased in *S. aureus-*induced apoptosis and necrosis and is regulated by YTHDF2 via m^6^A modification

**Figure 6:**
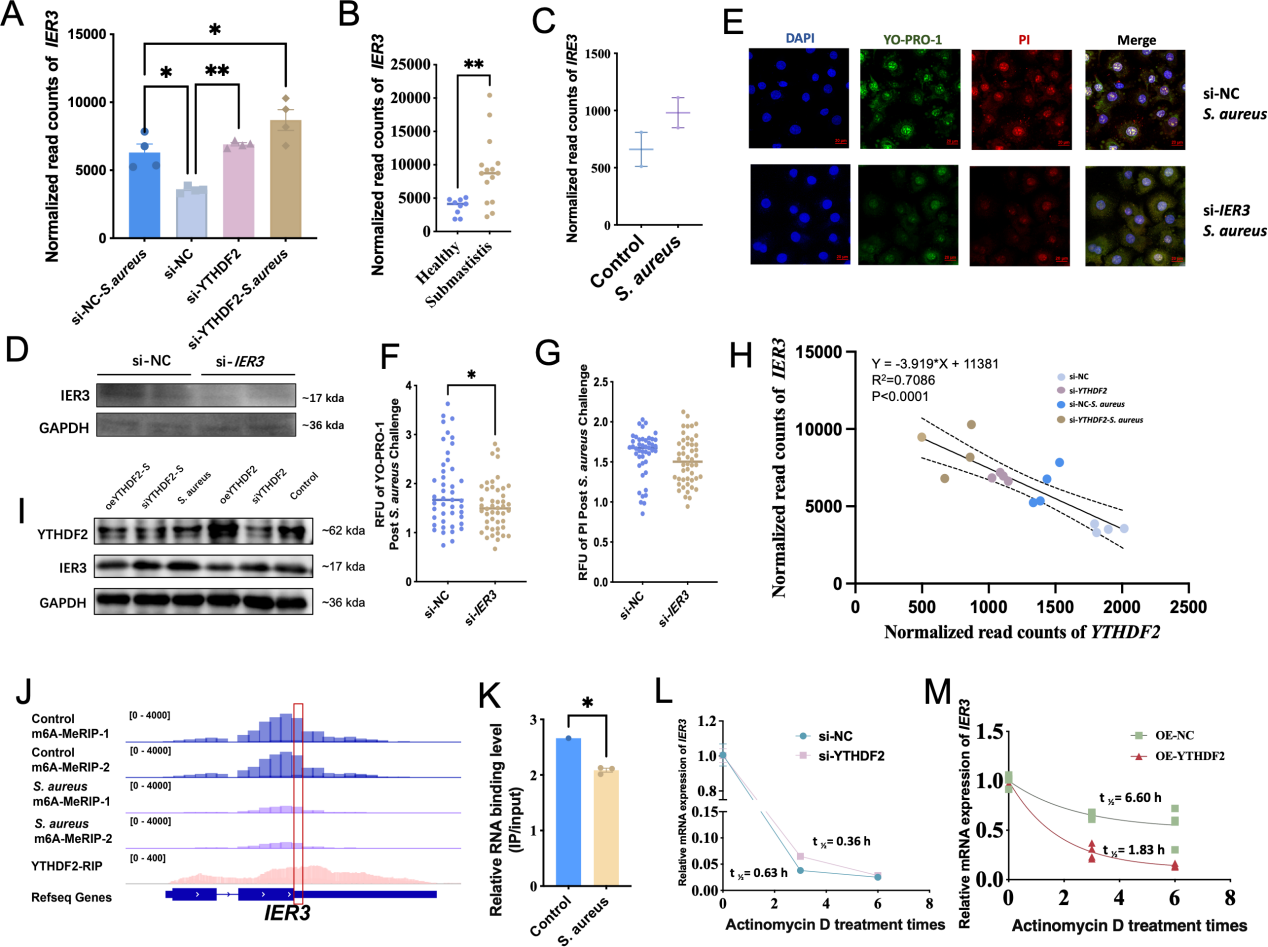
Regulation of *IER3* by YTHDF2 in Mac-T cells under *S. aureus* challenge. **(A)** Normalized *IER3* expression in Mac-T cells from different experimental groups: si-NC, si-YTHDF2, si-NC + *S. aureus*, and si-YTHDF2 + *S. aureus*. **(B)** Normalized *IER3* expression in milk samples from subclinical *S. aureus* mastitis cows and healthy cows. **(C)** Normalized *IER3* expression in mammary gland tissue samples from the bovine model. **(D)** WB analysis of IER3 expression in si-IER3 Mac-T cells. **(E-G)** Immunofluorescence images and relative fluorescence unit (RFU) quantification of YO-PRO-1 (green) and PI (red) in si-NC and si-*IER3* transfected Mac-T cells after *S. aureus* challenge. **(H)** Significant correlation between *IER3* gene expression and *YTHDF2* levels in Mac-T cells. **(I)** WB analysis showing the relationship between YTHDF2 and IER3 under different conditions. **(J)** m^6^A-MeRIP-seq data showing m^6^A modification peaks in the *IER3* gene in control and *S. aureus*-challenged Mac-T cells. **(K)** RIP-qPCR analysis showing m^6^A peaks and YTHDF2 binding sites in the 3’ UTR region of *IER3* under control and *S. aureus* conditions. **(L, M)** Half-life (t1/2) of *IER3* mRNA, illustrating its stability in Mac-T cells transfected with si-NC, si-YTHDF2, oe-NC, and oe-YTHDF2. Statistical significance is indicated as follows: *p < 0.05,**p < 0.01.

*IER3*, one of the 44 key genes regulated by YTHDF2, was found to be significantly upregulated during *S. aureus*-induced apoptosis and necrosis. *IER3* is a stress-responsive gene associated with apoptosis, as indicated by the GeneCards database (Stelzer et al., 2016). Normalized read counts further supported these findings, with IER3 expression significantly higher in YTHDF2 knockdown cells compared to controls (**Figure 6A**). Following *S. aureus* challenge, *IER3* expression was further increased in si-YTHDF2 cells, demonstrating that the absence of YTHDF2 amplifies *S. aureus*-induced upregulation of *IER3*. This upregulation was also observed in the transcriptomic data of milk samples from cows with subclinical mastitis and in blood transcriptomic data from cows infected with *S. aureus*, supporting the upregulation of IER3 during infection (**Figures 6B, C**).

To investigate the role of IER3 in apoptosis and necrosis, its expression was silenced in Mac-T cells. RT-qPCR and Western blot analyses confirmed efficient knockdown, with significant reductions in both *IER3* mRNA and protein levels (**Figure 6D**). Functional assays showed that *IER3* knockdown significantly reduced *S. aureus*-induced apoptosis and necrosis. YO-PRO-1/PI staining, visualized by confocal microscopy and quantified by a microplate reader, showed a significant decrease in apoptotic and necrotic cells in IER3 knockdown cells compared to controls (**Figure 6E-G**). These results suggest that IER3 acts as a pro-apoptotic factor during *S. aureus*-induced stress.

To further investigate the regulation of *IER3* by YTHDF2, a regression analysis was performed to examine the relationship between YTHDF2 and IER3 expression levels. A significant negative correlation was observed between the expression of *YTHDF2* and *IER3*, indicating that increased *YTHDF2* expression corresponds to decreased *IER3* expression (**Figure 6H**). Since YTHDF2 is known to promote mRNA degradation (Hou et al., 2021), this negative correlation suggests that YTHDF2 may regulate *IER3* through mRNA stability. Further experiments showed that si-YTHDF2 resulted in an increase in IER3 protein levels, while YTHDF2 overexpression resulted in a decrease in IER3 protein expression. Additionally, IER3 protein levels were significantly increased under *S. aureus* challenge conditions, indicating that YTHDF2 regulates IER3 in response to bacterial stress (**Figure 6I**).

To explore the mechanism by which YTHDF2 regulates *IER3*, SRAMP (Structural RNA Modification Annotation and Prediction, a tool for predicting RNA modifications based on sequence and structural features) was used to predict potential m^6^A modification sites in *IER3* mRNA. A high confidence m^6^A site was identified in the 3’ untranslated region of *IER3* (**Supplementary Figure S2E**). m^6^A-seq confirmed a significant reduction in the m^6^A peak of *IER3* mRNA following *S. aureus* challenge (**Figure 6J**). RIP-seq further validated that YTHDF2 binds to this m^6^A-modified site, confirming that YTHDF2 regulates *IER3* via m^6^A modification. Since YTHDF2 promotes mRNA degradation, the reduced m^6^A modification after *S. aureus* infection likely decreased YTHDF2 binding, slowed *IER3* degradation, and resulted in increased expression. RIP-qPCR results showed that reduced YTHDF2 binding after infection resulted in decreased *IER3* degradation and increased mRNA stability (**Figure 6K**). To further support this finding, actinomycin D assays were performed. The half-life (t1/2) of *IER3* mRNA increased from 0.36 hours to 0.63 hours in YTHDF2 knockdown cells, indicating increased mRNA stability in the absence of YTHDF2 (**Figure 6L**). In contrast, the mRNA half-life decreased from 6.59 hours to 1.83 hours in YTHDF2-overexpressing cells, confirming that YTHDF2 destabilizes *IER3* mRNA (**Figure 6M**). These results establish YTHDF2 as a critical regulator of *IER3* expression via m^6^A modification, which affects apoptosis and necrosis in *S. aureus*-challenged Mac-T cells.

## 4 Discussion

In previous studies, it has been widely demonstrated that m^6^A modifications are essential for regulating mRNA stability, degradation, and cellular processes like immune responses and apoptosis (Zhao et al., 2017; Huang et al., 2018; Uzonyi et al., 2023). This study used integrated approaches to investigate the m^6^A modification in the regulation of gene expression during *S. aureus* infection. First, RNA-seq analysis (siNC vs. siNC-*S. aureus*, siNC vs. siYTHDF2) identified infection-responsive transcripts and highlighted those potentially regulated by YTHDF2. This analysis facilitated the identification of genes altered during infection and those affected by YTHDF2. This analysis enabled the identification of genes altered during infection and those affected by YTHDF2. Subsequent analysis by m^6^A profiling revealed infection-specific differential methylation events, particularly in apoptosis-related genes such as *IER3*, thus providing insight into the role of m^6^A modification in the infection process. YTHDF2 RIP-seq was used to define its direct mRNA targets, and the integration of these data with m^6^A profiling identified infection-specific mRNAs that were directly bound by YTHDF2. These datasets were used to construct the m^6^A-YTHDF2-*IER3* pathway, thereby demonstrating that YTHDF2 regulates *IER3* mRNA degradation during *S. aureus* infection. These findings were further validated in subclinical mastitis and infected samples.

YTHDF2, an m^6^A “reader” protein, regulates mRNA stability by recognizing m^6^A modifications and promoting mRNA degradation (He et al., 2019; Chen et al., 2021). This mechanism is essential for controlling gene expression in various biological processes, including immune responses and apoptosis (Guo et al., 2020; Li et al., 2020; Einstein et al., 2021). *IER3* is an immediate-early response gene involved in cellular apoptosis and necrosis (Yoon et al., 2009; Ustyugova et al., 2012). During *S. aureus* infection, *IER3* upregulation has been observed to correlates with increased cell death, including apoptosis and necrosis, in Mac-T cells. This suggests that *IER3* not only plays a role in cellular stress responses but also contributes to regulating cell survival during infection. Knockdown of YTHDF2 (si-YTHDF2) resulted in a significant increase in *IER3* mRNA stability, as indicated by its prolonged half-life. In contrast, overexpression of YTHDF2 (oe-YTHDF2) resulted in a reduction in *IER3* mRNA stability. These findings highlight YTHDF2’s role in modulating IER3 stability through m^6^A modification, which is critical for immune response regulation during bacterial infection.

m^6^A modification sites, particularly within the 3’ UTR of mRNA, are essential for mRNA stability and are preferentially recognized by YTHDF2 (Guo et al., 2020; Li et al., 2021a). This study confirmed that YTHDF2 targets these specific m^6^A modification sites within *IER3* mRNA, regulating its stability and contributing to the fine-tuning of immune responses. Furthermore, YTHDF2 likely influences key inflammatory pathways, such as NF-κB and PI3K/Akt, which modulate apoptosis and immune responses (Arlt et al., 2003; Sina et al., 2010). By targeting IER3 mRNA, YTHDF2 not only regulates gene expression but also indirectly regulates the immune response and cell death pathways.

Of the 44 YTHDF2-regulated genes identified through m^6^A modifications, several are involved in immune responses and inflammation. This study confirmed that *IER3* is associated with apoptosis regulation, and its m^6^A-dependent modulation by YTHDF2 underscores the critical role of m^6^A in inflammatory processes of *S. aureus* challenge. Previous studies have extensively documented the pivotal role of m^6^A modifications in regulating immune responses and cellular stress pathways, affecting mRNA stability, translation, and splicing, which modulate key aspects of inflammation and immunity (Jiang et al., 2021; Mao et al., 2023; Zhu et al., 2023). Furthermore, GWAS enrichment analysis demonstrated significant associations between these genes and important bovine health traits, such as somatic cell score (Heringstad et al., 2006; Khan et al., 2023). This suggests that YTHDF2’s regulation of m^6^A-modified mRNAs not only impacts cellular responses to *S. aureus* infection but also influences broader health traits in dairy cattle, offering insights into genetic and epigenetic strategies for improving disease resistance.

Oxidative stress and mitochondrial dysfunction are critical mediators of apoptosis in Mac-T cells during *S. aureus* infection (Malhotra et al., 2019; Zhou et al., 2020). This study shows that knockdown of YTHDF2 significantly increases ROS levels, leading to mitochondrial dysfunction and enhanced apoptosis. These results are consistent with those of studies on bacterial infections, including those caused by *E. coli* (Jin et al., 2018; Zhang et al., 2023b) and *S. aureus* (Gao et al., 2017; Xu et al., 2022), where elevated ROS levels contribute to mitochondrial damage and apoptosis (Yang et al., 2016; Pinegin et al., 2018). Specifically, YTHDF2 knockdown exacerbates mitochondrial damage and apoptosis under *S. aureus* challenge, supporting the notion that YTHDF2 plays a protective role against oxidative damage. In contrast, YTHDF2 overexpression (oe-YTHDF2) mitigates these effects, preserving mitochondrial function and reducing apoptosis. This was evidenced by a lower JC-1 green/red fluorescence ratio in oe-YTHDF2 cells, indicating maintained mitochondrial membrane potential (MMP) and decreased ROS levels. These results suggest that YTHDF2 protects cells from oxidative stress by regulating m^6^A-modified mRNAs involved in oxidative stress responses, maintaining mitochondrial integrity and cellular health under stress.

Studies have shown that m^6^A modifications regulate the stability of mRNAs associated with oxidative stress and mitochondrial function (Sun et al., 2021; Yu et al., 2021; Wan et al., 2024). This study, along with ROS and JC-1 assays, further highlights the role of YTHDF2’s role in modulating mitochondrial function and ROS levels. By regulating m^6^A-modified mRNAs, YTHDF2 contributes to protecting cells from oxidative damage and apoptosis. This highlights how RNA modifications and their reader proteins influence cellular stress responses and mitochondrial function, ultimately impacting cell survival during bacterial infections.

This study provides valuable insights into the role of YTHDF2 in m^6^A modification, but several limitations should be addressed. First, the research primarily used the Mac-T cell model, which may not fully capture the complexity of cellular interactions in mammary tissue during infection. Future research could incorporate co-culture systems (e.g., Mac-T cells with mammary macrophages) (Yao et al., 2017; Spalinger et al., 2020) or 3D mammary gland tissue models to better simulate immune responses and tissue complexity (Lee et al., 2023). Another limitation is the small sample size, with only two cows tested, which limits the generalizability of the findings. Future studies should include a larger, more diverse sample of cows to confirm the robustness of the results. Additionally, this study primarily focused on the early immune response during *S. aureus* infection, leaving the chronic phase of mastitis underexplored. Future research should investigate the role of YTHDF2 and IER3 in chronic inflammation, providing a more complete understanding of long-term immune responses.

**Figure 7:**
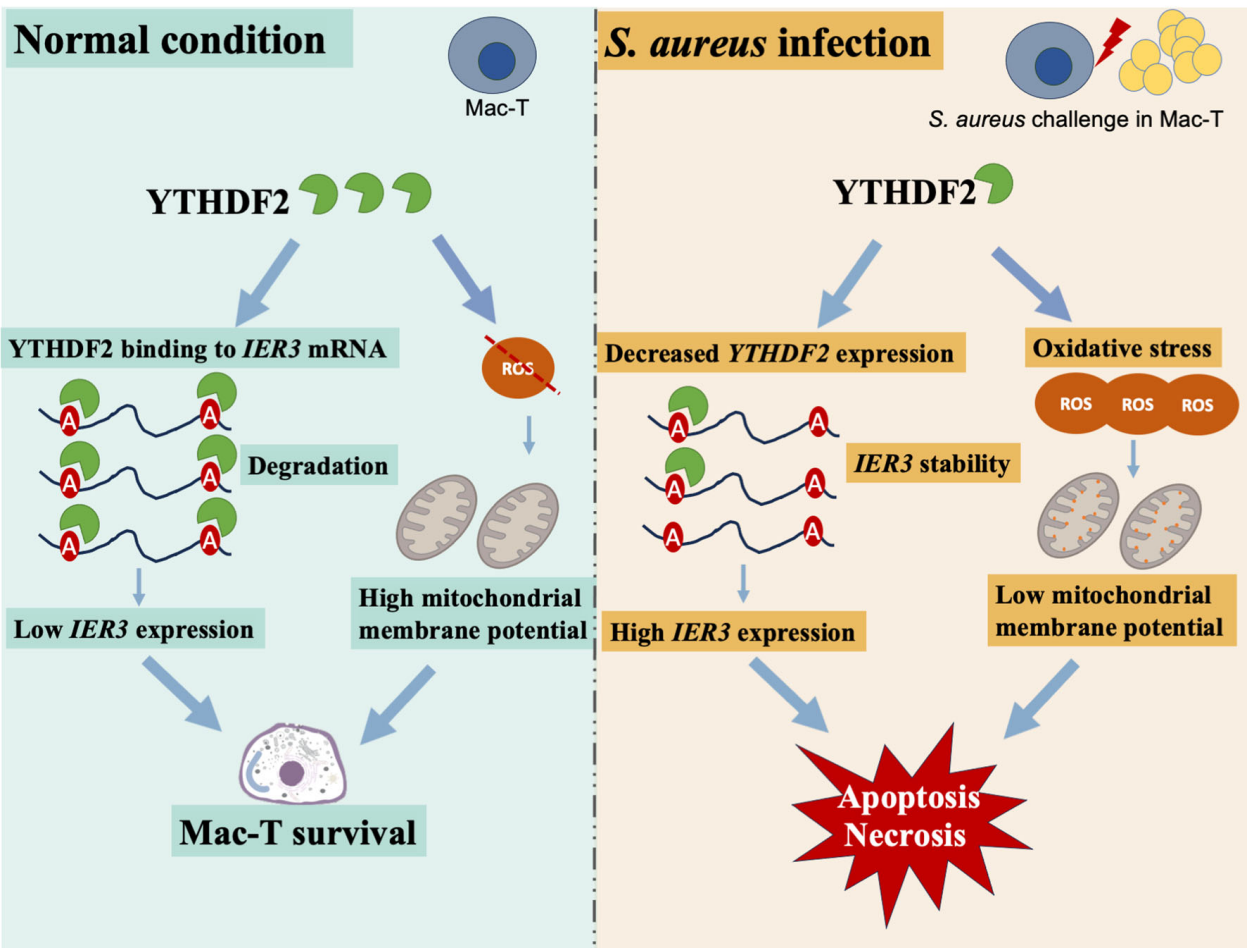
Mechanistic insights into YTHDF2 regulation of *IER3* mRNA stability and cell death in *S. aureus*-induced Mac-T cells.

Mastitis is a major challenge in the dairy industry, with *S. aureus* being one of the main pathogens responsible for it. This study shows that *S. aureus* infection decreases *YTHDF2* expression while increasing *IER3* expression in Mac-T cells, highlighting the role of YTHDF2 in regulating *IER3* mRNA stability through m^6^A modifications (**Figure 7**). Specifically, YTHDF2 has been shown to promote the degradation of m^6^A-modified *IER3* mRNA, and its downregulation has been observed to increase IER3 levels, which are associated with increased apoptosis and necrosis in infected cells. Furthermore, YTHDF2 plays a critical role in modulating apoptosis, mitochondrial function and ROS levels, thereby supporting cell survival during bacterial infection. In conclusion, this study highlights the essential role of YTHDF2 and IER3 in the inflammatory response of bovine mammary cells to *S. aureus* infection, providing novel insights into the molecular mechanisms underlying bovine mastitis. The findings of this study provide valuable implications for molecular breeding aimed at improving udder health in dairy cattle.

## Data Availability

The datasets presented in this study can be found in online repositories. The names of the repository/repositories and accession number(s) can be found in the article/**Supplementary Material**.

## Supplementary material

Supplementary Figure 1 KEGG pathway enrichment. **(A)** KEGG pathway enrichment analysis of DEGs post *S. aureus* challenge. **(B)** KEGG pathway enrichment of differentially expressed genes (DEGs) between treatment groups. **(C)** KEGG pathway enrichment of common DEGs across conditions.

Supplementary Figure 2 Effects of YTHDF2 knockdown on necrosis and m^6^A-modified gene regulation in Mac-T cells under *S. aureus* challenge. **(A)** Chromosomal distribution of transcription factor binding sites across the bovine genome. **(B)** Binding motif identified by HOMER showing the overlap between YTHDF2 RIP-seq and m^6^A-MeRIP-seq data. **(C)** Intersection analysis of gene sets from RIP-seq, JN-seq, and DE-m^6^A-seq, highlighting shared gene overlaps. **(D)** Venn diagram showing the overlap of DEGs among different experimental comparisons (N: si-NC, Y: si-*YTHDF2*, and JN: si-NC + *S. aureus*). **(E)** QTL associations with bovine traits, including disease susceptibility and production traits. **(F)** QTL enrichment analysis of the IER3 gene, showing associations with immunoglobulin G levels and disease resistance.

Supplementary Figure 3 Effects of YTHDF2 knockdown on necrosis and m^6^A-modified gene regulation in Mac-T cells under *S. aureus* challenge. **(A, B)** WB analysis showing YTHDF2 protein levels and relative gray values in Mac-T cells transfected with si-NC, si-YTHDF2, oe-NC and oe-YTHDF2. **(C)** Propidium iodide (PI) staining showing necrotic cells (red fluorescence) in si-NC and si-YTHDF2-transfected Mac-T cells post-*S. aureus* challenge. Dark field and bright field images are shown for comparison. **(D)** Correlation heatmap displaying expression relationships between m^6^A-modified DEGs regulated by YTHDF2. Significant correlations are indicated by P < 0.05. **(E)** Predicted m^6^A modification site on target mRNA, with the highlighted position located within the sequencing data obtained in this study. **(F)** Confidence score plot for predicted m^6^A sites across the transcript, showing regions of very high, high, moderate, and low confidence, with marked positions falling within the sequencing results from this study.
