# Supplementary material for: YTHDF2-mediated m6A modification regulates mRNA stability of Immediate early response gene 3 to modulate cell death in Staphylococcus aureus-induced bovine mastitis
Source: Front Cell Infect Microbiol. 2025 May 30;15:1542647. doi: 10.3389/fcimb.2025.1542647 (PMC12162568; doi:10.3389/fcimb.2025.1542647)

Supplement Table 1: Temperature and SCC measurements in cows

Time	Temperature (°C)		SCC (10^3 cells/ml)	
	cow#1	cow#2	cow#1	cow#2
-21 days	38.4	38.3	16	26
-3 days	38.4	38.5	12	12
0 h	38.5	38.2	16	29
6 h	40.8	41.0	5425	7196
12 h	41.2	41.3	8544	6992
18 h	41.4	41.5	9692	8743
24 h	41.8	41.5	8452	8232

Supplement Table 2: *S. aureus* virulence factor

GENE	*S. aureus* strain
cap8E	+
cap8G	+
cap8L	+
cap8O	+
cap8P	+
geh	+
hly/hla	+
lip	+
icaA	+
aur	+
hlgA	+
map	+
hlb	+
hld	+
adsA	+
cap8A	+
cap8B	+
cap8C	+
cap8D	+
cap8F	+
cap8N	+
esxA	+
esaA	+
esaB	+
esaC	+
esxB	+
isdB	+

isdA	+
isdC	+
isdE	+
isdF	+
isdG	+
icaC	+
icaB	+
icaD	+
icaR	+
sspC	+
sspA	+
ebp	+
sbi	+
hlgC	+
cap8M	+
essA	+
hysA	+
lukF-PV	+
srtB	+
fnbA	+
sspB	+
clfA	+
hlgB	+
coa	+
essB	+
isdD	+
fnbB	+
essC	+
spa	
sdrC	
cap8H	+
cap8I	+
cap8J	+
cap8K	+
sdrD	+
clfB	
sdrE	+
seh	+
vWbp	+

inhA
nheC
nheB
nheA
BAS319
lukS-PV
scn
chp
sak
sea

Supplement Table 3: Characterisation of homeostatic genes in *S.aureus*

Strain from cow	N50	ST	arcC	aroE	glpF	gmk	pta	tpi
Mastitis	317839	1	1	1	1	1	1	1

Supplement Table 4: Primer Sequences

Gene	Forward Primer (5'→3')	Reverse Primer (5'→3')
GAPDH	AGGTCGGAGTGAACGGATTC	CCAGCATCACCCCACTTGAT
YTHDF2	TCGGAGTCATATGCAGGTTCAG	CTTTTCGGACAGCACGAACG
IER3	TCCTCTACCCACGAGTGGTCC	TGCAGACGCACTGTCTTCAG
IER3-3'UTR	GCGAGAGCGTATCCCAAACT	TCACCTAGGGGGACACACAT

Supplement Table 5: Distribution of YTHDF2-bind mRNA peaks by chromosome

chromosome	RIP-peak number	percentage
1	451	4.4%
2	657	6.4%
3	501	4.9%
4	249	2.4%
5	605	5.9%
6	313	3.0%
7	461	4.5%
8	341	3.3%
9	205	2.0%
10	479	4.7%
11	483	4.7%
12	115	1.1%
13	324	3.2%
14	205	2.0%
15	390	3.8%
16	470	4.6%
17	236	2.3%

	18	492	4.8%
	19	561	5.5%
	20	219	2.1%
	21	414	4.0%
	22	288	2.8%
	23	370	3.6%
	24	102	1.0%
	25	357	3.5%
	26	146	1.4%
	27	71	0.6%
	28	154	1.5%
	29	374	3.6%
X		250	2.4%

Supplement Fig 1 KEGG pathway enrichment. (A) KEGG pathway enrichment analysis of DEGs post *S. aureus* challenge. **(B)** KEGG pathway enrichment of differentially expressed genes (DEGs) between treatment groups. **(C)** KEGG pathway enrichment of common DEGs across conditions.

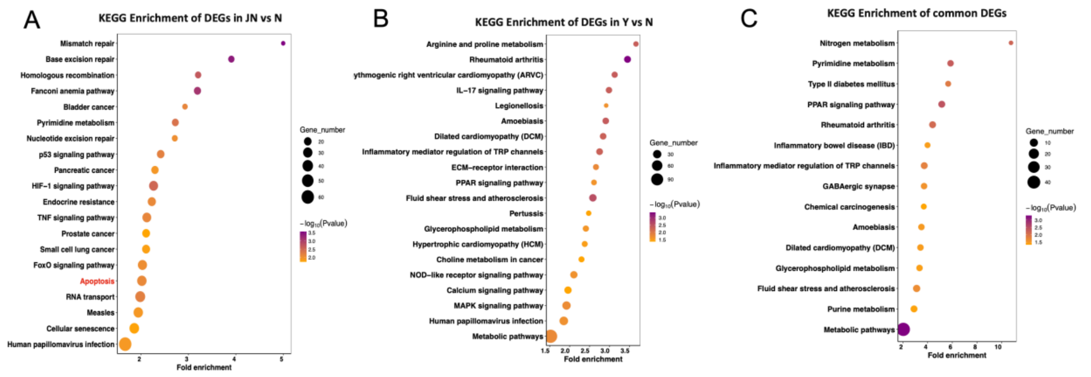

Supplement Fig 2 Genome-wide transcription factor binding distribution and QTL associations. (A) Chromosomal distribution of transcription factor binding sites across the bovine genome. **(B)** Binding motif identified by HOMER showing the overlap between YTHDF2 RIP-seq and m^{6}A-MeRIP-seq data. **(C)** Intersection analysis of gene sets from RIP-seq, JN-seq, and DE-m6A-seq, highlighting shared gene overlaps. **(D)** Venn diagram showing the overlap of DEGs among different experimental comparisons (N**:** si-NC, Y**:** si-*YTHDF2,* and JN**:** si-NC + *S. aureus*). **(E)** QTL associations with bovine traits, including disease susceptibility and production traits. **(F)** QTL enrichment analysis of the IER3 gene, showing associations with immunoglobulin G levels and disease resistance.

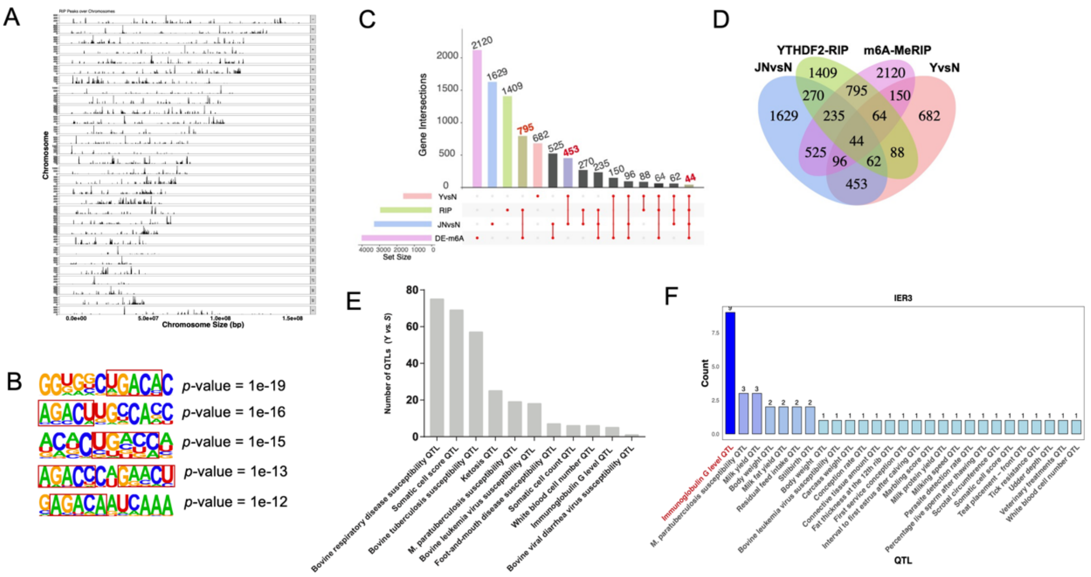

Supplement Fig 3 Effects of YTHDF2 knockdown on necrosis and m6A-modified gene

regulation in Mac-T cells under *S. aureus* challenge.

(A-B) WB analysis showing YTHDF2 protein levels and relative gray values in Mac-T cells transfected with si-NC, si-*YTHDF2*, oe-NC and oe-*YTHDF2*. **(C)** Propidium iodide (PI) staining showing necrotic cells (red fluorescence) in si-NC and si-YTHDF2-transfected Mac-T cells post-*S. aureus* challenge. Dark field and bright field images are shown for comparison. **(D)** Correlation heatmap displaying expression relationships between m6A-modified DEGs regulated by YTHDF2. Significant correlations are indicated by $P < 0.05$. **(E)** Predicted m6A modification site on target mRNA, with the highlighted position located within the sequencing data obtained in this study. **(F)** Confidence score plot for predicted m6A sites across the transcript, showing regions of very high, high, moderate, and low confidence, with marked positions falling within the sequencing results from this study.

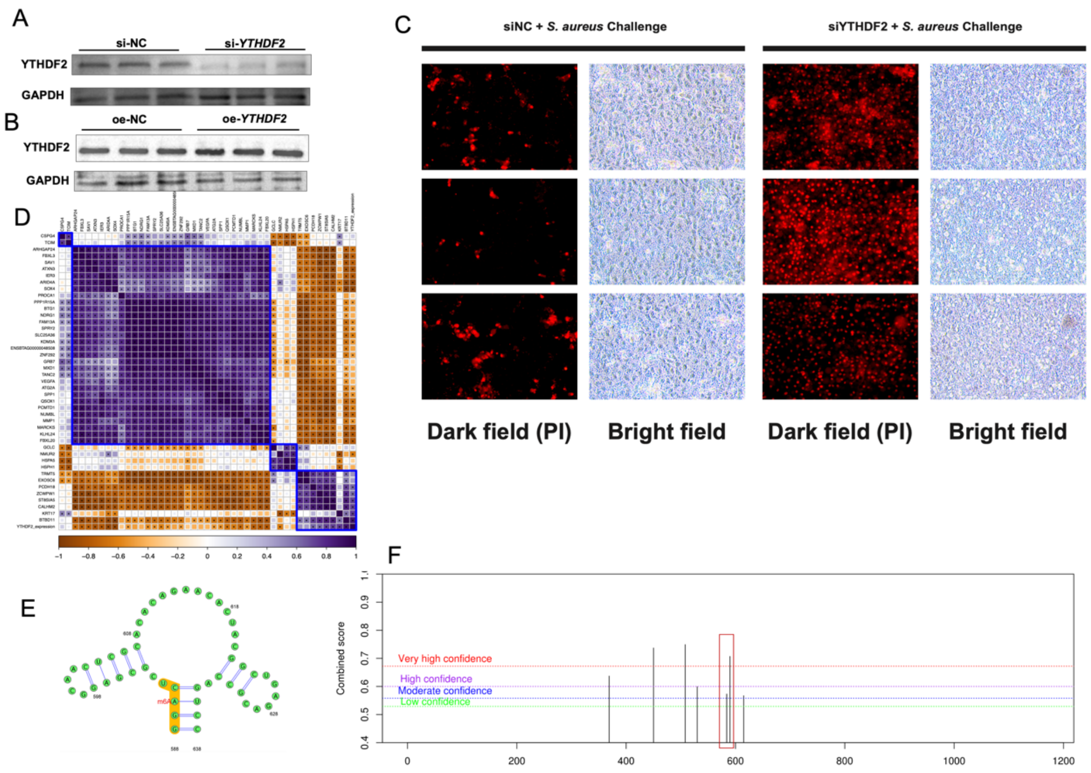

Supplement: Supplementary Figure 1 — KEGG pathway enrichment. (A) KEGG pathway enrichment analysis of DEGs post S. aureus challenge. (B) KEGG pathway enrichment of differentially expressed genes (DEGs) between treatment groups. (C) KEGG pathway enrichment of common DEGs across conditions. [file DataSheet1.pdf]
